# The Evolution of Machine Learning and Its Applications in Orthopaedics: A Bibliometric Analysis

**DOI:** 10.7759/cureus.95296

**Published:** 2025-10-24

**Authors:** Panagiotis Bompolas, Sina Dehnadi, Senthooran Kathiravelupillai, Aasim Hagroo, Kiamehr Karagah

**Affiliations:** 1 Trauma and Orthopaedics, Buckinghamshire Healthcare NHS Trust, Aylesbury, GBR; 2 Accident and Emergency, Royal Free Hospital NHS Foundation Trust, London, GBR; 3 Surgery, James Paget University Hospital, Gorleston, GBR; 4 Trauma and Orthopaedics, Queen Elizabeth Hospital, London, GBR; 5 College of Letters and Science, University of California Berkeley, Berkeley, USA

**Keywords:** artificial intelligence, chatgpt, convolutional neural networks, deep learning, machine learning, orthopaedics

## Abstract

Artificial intelligence (AI) and machine learning (ML) are computational systems designed to perform tasks that typically require human intelligence, with the capability to learn and improve by processing real-time data. Ongoing advancements in these models have led to their growing application in the field of medicine, leveraging their capabilities to enhance outcomes. To explore their impact in orthopaedics, the 100 most-cited articles on ML applications were identified through a comprehensive search of all databases within Web of Science, limited to English-language publications but with no restriction on publication year. Data were extracted to analyse distinct key aspects of ML methodology and applications within different orthopaedic subspecialties. The level of evidence (LoE) of included studies was also assessed.

The included articles collectively accounted for a total of 10,886 citations. Citation count per article ranged significantly from 57 to 428 (mean: 108.9 ± 56.1). The majority of the studies were classified as LoE V (n = 46; mean citations = 108 ± 41.9), with 43 of them being experimental in terms of study design. Only one study achieved level I status, highlighting a significant gap in methodological quality research within the field. Musculoskeletal imaging was the most prominently represented subspecialty (n = 44), followed by trauma (n = 23) and arthroplasty (n = 21). Convolutional neural networks (CNNs) were predominant in terms of ML technique (n = 37), while deep learning (DL) was the most common ML field discussed. A total of 17% of studies included a human comparison group, with AI in orthopaedics generally demonstrating performance close to, but seldom surpassing, that of human experts. ChatGPT (versions 3.5 and 4.0) did not demonstrate superior performance compared to orthopaedic surgeons in four separate studies where direct comparisons were made.

Overall, most of the highly influential articles on machine learning applications in orthopaedics are based on lower levels of evidence. These models require more critical evaluation and strong human oversight to ensure their effective integration into routine orthopaedic practice and to support a productive collaboration between humans and AI systems.

## Introduction and background

Artificial intelligence (AI) refers to a machine's ability to perform tasks that typically require human intelligence, such as speech recognition, natural language understanding, and decision-making. Within AI, machine learning (ML) is a subset that uses algorithms to automate decision-making by relying on models trained on data, rather than being explicitly programmed [1,2]. As such, there is a large scope for the application of both within the context of medicine and surgery, where they could help reduce the administrative burden that physicians encounter or act as decision-support tools that can optimise clinical and patient-reported outcomes, such as predicting coronary heart disease or classifying diabetic retinopathy severity [3,4]. 

That said, AI and ML have had a growing impact on patient management in orthopaedics worldwide, with an average annual growth rate of 19.45% since 1988 [5]. Advancements in these technologies, along with the development of models such as convolutional neural networks (CNNs), generative adversarial networks (GANs), and artificial neural networks (ANNs), have driven the ongoing integration of AI and ML into medicine and surgery, through their abilities to recognise patterns, generate new data, and learn from input data. Consequently, their performance in key tasks often surpasses that of humans [6-8]. Such tasks have been predominantly relevant to imaging, which is integral to diagnosis and treatment within orthopaedics. However, as orthopaedic care becomes more complex and evidence-based practice is more widely adopted, understanding and integrating these technologies is essential to enhance efficiency and improve outcomes, especially in areas like early fracture detection, surgical planning, implant survivorship prediction, and personalised rehabilitation.

Although prior studies have assessed the impact of AI in orthopaedics and analysed ML trends specifically in arthroplasty, none have explored the combined use of AI and ML throughout the entire field of orthopaedic surgery and musculoskeletal medicine [1,9]. This gap in research limits our understanding of how AI and ML have evolved and been integrated into different orthopaedic subspecialties. AI, combined with deep learning (DL), has demonstrated its ability to assist physicians by, for example, highlighting suspicious regions in images for radiologists and pathologists to review, thereby enabling focused and specific task support [10]. Current clinical applications are limited to three categories: predictive analytics (using patient data to forecast outcomes or complications), computer vision (analysing medical images such as X-rays or MRIs) and natural language processing (NLP - interpreting information from clinical notes or reports). Future directions for ML models involve the development of adaptive techniques and the integration of real-time data processing to train and refine their algorithms, allowing them to continuously update in line with advancements in medicine and surgery [11]. 

Bibliometric analyses offer a systematic evaluation of the scientific literature, providing insights into its growth, impact, emerging trends and existing gaps [12,13]. Analysis of article citation patterns affects the authors' and their affiliated institutions' reputation, as well as the publishing journal's impact factor (IF). Additionally, the quality of a study’s design is typically categorised by a specific level of evidence, which reflects its methodological rigour, reproducibility of outcomes, and overall reliability. With that in mind, this study represents the first bibliometric analysis focused on the application of ML across the entire field of orthopaedic surgery and the musculoskeletal system, specifically focusing on the 100 most-cited articles globally. It aims to evaluate the evolution of ML in the field, analyse the use of various ML models and performance metrics, investigate the interaction between ML systems and human practitioners, and identify trends and gaps in the literature, while also addressing emerging challenges related to data quality, bias, human oversight, and clinical validation. Ultimately, it aims to aid clinicians, researchers and policymakers to better understand the field’s trajectory and guide future efforts.

## Review

Materials and methods

Search Strategy

In July 2025, a comprehensive search was conducted across all databases available in the Web of Science using a topic-based strategy to identify publications related to ML in orthopaedics. The search was performed utilising the following terms and Boolean operators: TS=(("artificial intelligence" OR "AI" OR "machine learning" OR "deep learning" OR "neural network*" OR "computer vision" OR "predictive modelling" OR "automated decision support" OR "AI-driven analysis" OR "data-driven algorithm*") AND ("orthop?edic* surgery" OR "orthop?edic*" OR "reconstructive surgery" OR "hand surgery" OR "ortho surgery")). The search was limited to articles in the English language.

**Figure 1:**
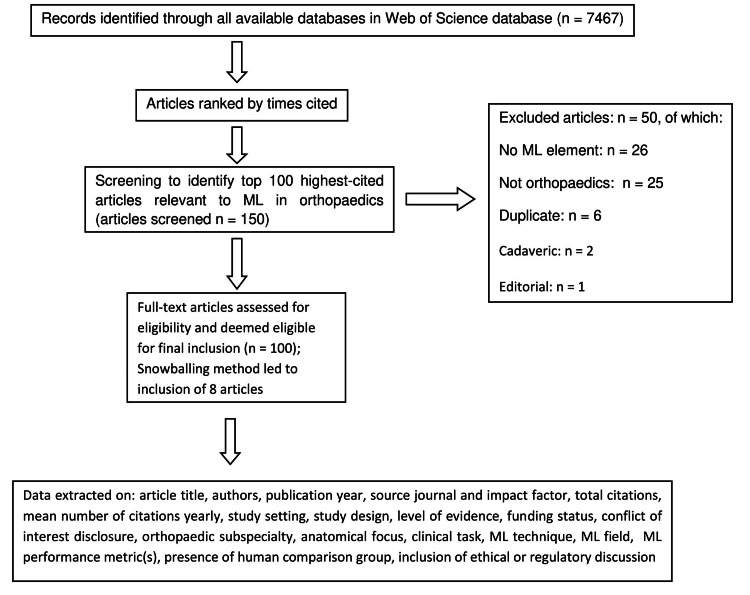
Flowchart depicting the methodology and search process with reasons for exclusion ML: machine learning

The top 100 most cited articles from the final dataset were identified and analysed for bibliometric trends. Figure 1 presents a summary of the methodology as well as reasons for exclusion. The snowballing method was employed to further identify relevant articles and evaluate their suitability for inclusion, leading to the addition of eight articles through this approach.

The search yielded a total of 7,467 articles. When articles had the same number of citations, they were ranked according to their average citations per year, with more recent publications placed higher. Two authors (P.B., K.G.) independently, manually, screened titles and abstracts until 100 articles directly relevant to ML in orthopaedics were identified. Any inconsistencies were addressed via consultation with a third author (S.D.) and by examining the complete text of the publication.

Data extraction of the full-text articles was performed manually and subsequently analysed via the Jamovi statistical software (version 2.6), a free and open-source software [14]. All analyses performed were descriptive in nature, with no inferential or advanced statistical synthesis being performed, consistent with a bibliometric analysis methodology. The level of evidence was assessed according to the Oxford Centre for Evidence-based Medicine [15].

Results

Distribution of Citations

The 100 top-cited articles related to the application of ML in orthopaedics and the musculoskeletal system amassed a total of 10,886 references, with a mean citation count of 108.9 ± 56.1 per article (range: 57-428), as outlined in Table 1 [16-115]. Additionally, the mean number of citations per article per year was 17.4 ± 15.6 (range: 2.3-105).

**Table 1:** Characteristics of the top 100 most-cited articles relevant to ML applications in orthopaedics, ranked in the descending order of total citation count

Title	Authors	Year	Total citation count	Mean citation count per year	AI/ML technique	Key outcomes/tasks
Deep neural network improves fracture detection by clinicians [16]	Lindsey R, Daluiski A, Chopra S, et al.	2018	428	61.1	Convolutional neural network (CNN)	Diagnosis
Artificial intelligence in fracture detection: transfer learning from deep convolutional neural networks [17]	Kim DH, MacKinnon T	2018	338	48.3	Convolutional neural network (CNN)	Diagnosis
Automated segmentation of knee bone and cartilage combining statistical shape knowledge and convolutional neural networks: data from the Osteoarthritis Initiative [18]	Ambellan F, Tack A, Ehlke M, Zachow S	2019	261	43.5	Convolutional neural network (CNN), statistical shape models (SSM)	Image segmentation
Iterative fully convolutional neural networks for automatic vertebra segmentation and identification [19]	Lessmann N, van Ginneken B, de Jong PA, Išgum I	2019	209	34.8	Convolutional neural network (CNN)	Image segmentation, diagnosis, education/training, research
LOGISMOS-Layered Optimal Graph Image Segmentation of Multiple Objects and Surfaces: cartilage segmentation in the knee joint [20]	Yin Y, Zhang X, Williams R, Wu X, Anderson DM, Sonka M	2010	203	13.5	Random forest	Surgical planning, research, image segmentation, outcome analysis
Computer-assisted bone age assessment: image preprocessing and epiphyseal/metaphyseal ROI extraction [21]	Pietka E, Gertych AP, Pospiech S, Cao F, Huang HZ, Gilsanz V	2001	186	7.8	Clustering	Diagnosis, outcome prediction, decision support, research
Detecting intertrochanteric hip fractures with orthopedist-level accuracy using a deep convolutional neural network [22]	Urakawa T, Tanaka Y, Goto S, Matsuzawa H, Watanabe K, Endo N	2018	181	25.9	Convolutional neural network (CNN)	Diagnosis
Trainable rule-based algorithm for the measurement of joint space width in digital radiographic images of the knee [23]	Duryea J, Li J, Peterfy CG, Gordon C, Genant HK	2000	170	6.8	Other	Diagnosis, outcome prediction, research, decision support
Cross-Modality Image Synthesis from Unpaired Data Using CycleGAN Effects of Gradient Consistency Loss and Training Data Size [24]	Hiasa Y, Otake Y, Takao M, et al.	2018	168	24	Generative adversarial network (GAN), convolutional neural network (CNN)	Image segmentation, diagnosis, decision support, research
Deep convolutional neural network for segmentation of knee joint anatomy [25]	Zhou Z, Zhao G, Kijowski R, Liu F	2018	166	23.7	Convolutional neural network (CNN)	Image segmentation
3D/2D registration and segmentation of scoliotic vertebrae using statistical models [26]	Benameur S, Mignotte M, Parent S, Labelle H, Skalli W, de Guise J	2003	158	7.2	Statistical shape models (SSM)	Image segmentation
Vertebrae localisation in pathological spine CT via dense classification from sparse annotations [27]	Glocker B, Zikic D, Konukoglu E, Haynor DR, Criminisi A	2020	157	31.4	Random forest, convolutional neural network (CNN)	Diagnosis, surgical planning
Can machine learning algorithms predict which patients will achieve minimally clinically important differences from total joint arthroplasty? [28]	Fontana MA, Lyman S, Sarker GK, Padgett DE, MacLean CH	2019	154	25.7	Logistic regression, random forest, supervised vector machine (SVM)	Outcome prediction, decision support, research
2D-3D shape reconstruction of the distal femur from stereo X-ray imaging using statistical shape models [29]	Baka N, Kaptein BL, de Bruijne M, et al.	2011	150	10.7	Statistical shape models (SSM), principal component analysis (PCA)	Image segmentation, surgical planning, outcome analysis, research
Neural network control of functional neuromuscular stimulation systems: computer simulation studies [30]	Abbas JJ, Chizeck HJ	1995	147	4.9	Artificial neural network (ANN), fuzzy neural network (FNN)	Surgical planning, outcome prediction, decision support, research
Machine learning in control of functional electrical stimulation systems for locomotion [31]	Kostov A, Andrews BJ, Popovic DB, Stein RB, Armstrong WW	1995	147	4.9	Artificial neural network (ANN), supervised learning	Decision support, surgical planning, outcome prediction, research
An upper-limb power-assist exoskeleton using proportional myoelectric control [32]	Tang Z, Zhang K, Sun S, Gao Z, Zhang L, Yang Z	2014	144	13.1	Artificial neural network (ANN)	Diagnosis, outcome prediction, decision support, research
Predicting 90-day and 1-year mortality in spinal metastatic disease: development and internal validation [33]	Karhade AV, Thio QCBS, Ogink PT, et al.	2019	142	23.7	Convolutional neural network (CNN), supervised vector machine (SVM), random forest, logistic regression, superior gradient boosting (SGB)	Outcome prediction
Patient-specific ankle-foot orthoses using rapid prototyping [34]	Mavroidis C, Ranky RG, Sivak ML, et al.	2011	139	9.9	No information available	Surgical planning, decision support, outcome analysis
Artificial intelligence and orthopaedics: an introduction for clinicians [35]	Myers TG, Ramkumar PN, Ricciardi BF, Urish KL, Kipper J, Ketonis C	2020	138	27.6	Convolutional neural network (CNN), supervised vector machine (SVM), random forest, logistic regression, artificial neural network (ANN), natural language processing (NLP), clustering, supervised learning, no information available	Image segmentation, diagnosis, outcome prediction, decision support, research, education/training, and surgical planning
Knee x-ray image analysis method for automated detection of osteoarthritis [36]	Shamir L, Ling SM, Scott WW, et al.	2009	133	8.3	K-nearest neighbours (KNN), other	Diagnosis, decision support, research
Instantiation and registration of statistical shape models of the femur and pelvis using 3D ultrasound imaging [37]	Barratt DC, Chan CSK, Edwards PJ, et al.	2008	132	7.8	Principal component analysis (PCA)	Surgical planning, decision support, research
Intraoperative image-based multiview 2D/3D registration for image-guided orthopaedic surgery: incorporation of fiducial-based C-arm tracking and GPU-acceleration [38]	Otake Y, Armand M, Armiger RS, et al.	2012	131	10.1	Supervised learning, no information available	Image segmentation, surgical planning, decision support, research
Augmented and virtual reality in spine surgery, current applications and future potentials [39]	Ghaednia H, Fourman MS, Lans A, et al.	2021	130	32.5	No information available	Surgical planning, decision support, education/training
Computer-aided method for quantification of cartilage thickness and volume changes using MRI: validation study using a synthetic model [40]	Kauffmann C, Gravel P, Godbout B, et al.	2003	129	5.9	Other	Image segmentation, diagnosis, surgical planning, outcome prediction, decision support, research
Atlas-based segmentation of degenerated lumbar intervertebral discs from MR images of the spine [41]	Michopoulou SK, Costaridou L, Panagiotopoulos E, Speller R, Panayiotakis G, Todd-Pokropek A	2009	128	8	Ensemble, clustering, fuzzy C-means (FCM)	Image segmentation, diagnosis, surgical planning, decision support, research
Scalable muscle-actuated human simulation and control [42]	Lee S, Park M, Lee K, Lee J	2019	126	21	Artificial neural network (ANN), supervised learning, deep reinforcement learning (DRL)	Surgical planning, outcome prediction, education/training, research
Automated comprehensive adolescent idiopathic scoliosis assessment using MVC-Net [43]	Wu H, Bailey C, Rasoulinejad P, Li S	2018	126	18	Convolutional neural network (CNN), supervised learning	Diagnosis, outcome prediction, decision support, research
3D multi-scale FCN with random modality voxel dropout learning for intervertebral disc localisation and segmentation from multi-modality MR Images [44]	Li X, Dou Q, Chen H, et al.	2018	126	18	Fully convolutional network (FCN), convolutional neural network (CNN), supervised learning	Image segmentation, outcome analysis, decision support, localisation
3D/2D registration and segmentation of scoliotic vertebrae using statistical models [45]	Benameur S, Mignotte M, Parent S, Labelle H, Skalli W, de Guise J	2003	125	5.7	No information available	Diagnosis
Automated 3-D PDM construction from segmented images using deformable models [46]	Kaus MR, Pekar V, Lorenz C, Roel Truyen, S. Lobregt, Weese J	2003	125	5.7	Principal component analysis (PCA), statistical shape models (SSM)	Image segmentation, diagnosis
How to build a patient-specific hybrid simulator for orthopaedic open surgery: benefits and limits of mixed-reality using the Microsoft HoloLens [47]	Condino S, Turini G, Parchi PD, et al.	2018	122	17.4	Other	Education/training
What are the applications and limitations of artificial intelligence for fracture detection and classification in orthopaedic trauma imaging? A systematic review [48]	Langerhuizen DWG, Janssen SJ, Mallee WH, et al.	2019	121	20.2	Convolutional neural network (CNN), supervised vector machine (SVM), Artificial neural network (ANN), random forest, other	Image segmentation, diagnosis, decision support, and other
Assessing ChatGPT responses to common patient questions regarding total hip arthroplasty [49]	Mika A, J. Ryan Martin, Engstrom SM, Polkowski GG, Wilson JM	2023	118	59	Natural language processing (NLP)	Education/training
Development of machine learning algorithms for the prediction of 30-day mortality after surgery for spinal metastasis [50]	Karhade AV, Thio QCBS, Ogink PT, et al.	2018	118	16.9	Convolutional neural network (CNN), supervised vector machine (SVM), random forest, decision tree, Bayes point machine (BPM)	Outcome prediction
Bone morphing: 3D morphological data for total knee arthroplasty [51]	Stindel É, Briard JL, Merloz P, et al.	2002	117	5.1	Statistical shape models (SSM)	Surgical planning
A neuro-control system for the knee joint position control with quadriceps stimulation [52]	Chang GC, Lub JJ, Liao GD, et al.	1997	115	4.1	Artificial neural network (ANN)	Research
Deep learning in fracture detection: a narrative review [53]	Kalmet PHS, Sanduleanu S, Primakov S, et al.	2020	113	22.6	Convolutional neural network (CNN)	Diagnosis
Machine learning and conventional statistics: making sense of the differences [54]	Ley C, Martin RK, Pareek A, Groll A, Seil R, Tischer T	2022	112	37.3	Supervised learning, Decision tree, supervised vector machine (SVM)	Diagnosis, outcome analysis, outcome prediction
Computer-assisted interpretation of planar whole-body bone scans [55]	Sadik M, Hamadeh I, Nordblom P, et al.	2008	112	6.6	Artificial neural network (ANN)	Diagnosis, outcome analysis
Performances of hill-type and neural network muscle models-toward a myosignal-based exoskeleton [56]	Rosen J, Fuchs MB, Arcan M	1999	109	4.2	Artificial neural network (ANN)	Research
Applying densely connected convolutional neural networks for staging osteoarthritis severity from plain radiographs [57]	Norman B, Pedoia V, Noworolski A, Link TM, Majumdar S	2024	106	15	GPT	Research
Prompt engineering in consistency and reliability with the evidence-based guideline for LLMs [58]	Wang L, Chen X, Deng X, et al.	2018	105	105	Other	Diagnosis
Automatic bone age assessment for young children from newborn to 7-year-old using carpal bones [59]	Zhang A, Gertych A, Liu BJ	2018	105	15	Convolutional neural network (CNN)	Diagnosis
Deep learning for detection of complete anterior cruciate ligament tear [60]	Chang PD, Wong TT, Rasiej MJ	2019	104	17.3	Convolutional neural network (CNN), other	Diagnosis
Incorporating a statistically based shape model into a system for computer-assisted anterior cruciate ligament surgery [61]	Fleute M, Lavallée S, Julliard R	1999	102	3.9	Principal component analysis (PCA)	Surgical planning, decision support, research
Label-free intraoperative histology of bone tissue via deep-learning-assisted ultraviolet photoacoustic microscopy [62]	Cao R, Nelson SD, Davis S, et al.	2023	99	49.5	Generative adversarial network (GAN)	Diagnosis, surgical planning
Automated bone mineral density prediction and fracture risk assessment using plain radiographs via deep learning [63]	Hsieh Chen-I, Zheng K, Lin C, et al.	2021	99	24.8	Convolutional neural network (CNN)	Diagnosis
Vertebral shape: automatic measurement with active shape models [64]	Smyth PP, Taylor CJ, Adams JE	1997	98	3.5	Other	Diagnosis
Machine learning for prediction of sustained opioid prescription after anterior cervical discectomy and fusion [65]	Karhade AV, Ogink PT, Thio QCBS, et al.	2019	96	16	Supervised vector machine (SVM), random forest, logistic regression, convolutional neural network (CNN), superior gradient boosting (SGB)	Outcome prediction
Automated detection, 3D segmentation and analysis of high-resolution spine MR images using statistical shape models [66]	Neubert A, Fripp J, Engstrom C, et al.	2012	95	7.3	Statistical shape models (SSM)	Image segmentation
Automated identification of anatomical landmarks on 3D bone models reconstructed from CT scan images [67]	Subburaj K, Ravi B, Agarwal M	2009	92	5.8	Convolutional neural network (CNN)	Surgical planning, diagnosis, and outcome prediction
Feature extraction using an RNN autoencoder for skeleton-based abnormal gait recognition [68]	Jun K, Lee DW, Lee K, Lee S, Kim MS	2020	91	18.2	Convolutional neural network (CNN)	Diagnosis
Assessment of a deep-learning system for fracture detection in musculoskeletal radiographs [69]	Jones RM, Sharma A, Hotchkiss R, et al.	2020	89	17.8	Convolutional neural network (CNN)	Diagnosis
Fully automatic cervical vertebrae segmentation framework for X-ray images [70]	Masudur Rahman AASM, Knapp K, Slabaugh G	2018	89	12.7	Convolutional neural network (CNN)	Image segmentation
Comparison of ChatGPT-3.5, ChatGPT-4, and orthopaedic resident performance on orthopaedic assessment examinations [71]	Massey PA, Montgomery C, Zhang AS	2023	88	44	Natural language processing (NLP)	Diagnosis
Can artificial intelligence pass the American Board of Orthopaedic Surgery examination? Orthopaedic residents versus ChatGPT [72]	Lum ZC	2023	87	43.5	Natural language processing (NLP)	Diagnosis, decision support
Robust statistical shape models for MRI bone segmentation in the presence of a small field of view [73]	Schmid J, Kim J, Magnenat-Thalmann N	2010	86	5.7	Artificial neural network (ANN), adaptive logic network (ALN)	Decision support, surgical planning, outcome analysis
Screening of knee-joint vibroarthrographic signals using statistical parameters and radial basis functions [74]	Rangayyan RM, Wu YF	2008	85	5	Artificial neural network (ANN)	Diagnosis
3D printed biomedical devices and their applications: a review on state-of-the-art technologies, existing challenges, and future perspectives [75]	Mamo HB, Adamiak M, Kunwar A	2023	82	41	Logistic regression	Research
Machine learning in knee osteoarthritis: a review [76]	Kokkotis C, Moustakidis S, Papageorgiou E, Giakas G, Tsaopoulos DE	2020	82	16.4	Convolutional neural network (CNN), supervised vector machine (SVM), random forest, long-short term memory (LSTM), ensemble, logistic regression	Research
Automated segmentation of the acetabulum and femoral head from 3-D CT images [77]	Zoroofi RA, Sato Y, Sasama T, et al.	2003	81	3.7	Other	Image segmentation, surgical planning, decision support
Development of machine learning algorithms for the prediction of prolonged opioid prescription after surgery for lumbar disc herniation [78]	Karhade AV, Ogink PT, Thio S, et al.	2019	80	13.3	Convolutional neural network (CNN), supervised vector machine (SVM), random forest, logistic regression	Outcome prediction
Deep learning to segment pelvic bones: large-scale CT datasets and baseline models [79]	Liu P, Han H, Du Y, et al.	2021	79	19.8	Supervised learning	Image Segmentation
Automatic grading of individual knee osteoarthritis features in plain radiographs using deep convolutional neural networks [80]	Tiulpin A, Saarakkala S	2020	79	15.8	Convolutional neural network (CNN), ensemble	Diagnosis
Cascaded statistical shape model-based segmentation of the full lower limb in CT [81]	Audenaert E, Houcke JV, Almeida D, et al.	2019	79	13.2	Statistical shape models (SSM)	Image segmentation
Predicting patient-reported outcomes following hip and knee replacement surgery using supervised machine learning [82]	Huber M, Kurz C, Leidl R	2019	78	13	Supervised learning, random forest, K-nearest neighbours (KNN), logistic regression, other	Outcome prediction
A scalable physician-level deep learning algorithm detects universal trauma on pelvic radiographs [83]	Cheng C, Wang Y, Chen H, et al.	2021	76	19	Supervised learning	diagnosis
Can artificial intelligence improve the readability of patient education materials [84]	Kirchner GJ, Kim RY, Weddle J, Bible JE	2023	75	37.5	GPT	Education/training
Deep convolutional neural network-based segmentation and classification of difficult-to-define metastatic spinal lesions in 3D CT data [85]	Chmelik J, Jakubicek R, Walek P, et al.	2018	74	10.6	Convolutional neural network (CNN), random forest	Image segmentation
Fully automated segmentation of cartilage from the MR images of the knee using a multi-atlas and local structural analysis method [86]	Lee JG, Gumus S, Moon CH, Kwoh CK, Bae KT	2014	74	6.7	Statistical shape models (SSM)	Image segmentation
Can machine-learning techniques be used for 5-year survival prediction of patients with chondrosarcoma? [87]	Thio S, Karhade AV, Ogink PT, et al.	2018	73	10.4	Convolutional neural network (CNN), supervised vector machine (SVM), Bayes point machine (BPM), decision tree	Outcome prediction
Neural-network-based nonlinear model predictive tracking control of a pneumatic muscle actuator-driven exoskeleton [88]	Cao Y, Huang J	2020	73	14.6	Artificial neural network (ANN)	Research
Advanced deep learning techniques applied to automated femoral neck fracture detection and classification [89]	Mutasa S, Varada S, Goel A, Wong TT, Rasiej MJ	2020	71	14.2	Convolutional neural network (CNN), generative adversarial network (GAN)	Diagnosis
SLIDE: automatic spine level identification system using a deep convolutional neural network [90]	Hetherington J, Lessoway V, Gunka V, Abolmaesumi P, Rohling R	2017	71	8.9	Convolutional neural network (CNN)	Diagnosis
Development of a statistical shape model of the patellofemoral joint for investigating relationships between shape and function [91]	Fitzpatrick CK, Baldwin MA, Laz PJ, FitzPatrick DP, Lerner LA, Rullkoetter PJ	2011	71	5.1	Statistical shape models (SSM)	Research, image segmentation
Optimisation of orthopaedic implant design using statistical shape space analysis based on level sets [92]	Kozic N, Weber S, Philippe Büchler, et al.	2010	70	4.7	Statistical shape models (SSM), principal component analysis (PCA)	Surgical planning
Automated 3D geometric reasoning in computer-assisted joint reconstructive surgery [93]	Subburaj K, Ravi B, Agarwal M	2009	70	4.4	Other	Image segmentation, surgical planning
Integration of computer-assisted bone age assessment with clinical PACS [94]	Pietka E, Pospiech-Kurkowska S, Gertych A, Cao F	2003	70	3.2	Clustering, fuzzy neural network (FNN)	Image segmentation
Machine learning methods to support personalised neuromusculoskeletal modelling [95]	Saxby DJ, Killen BA, Pizzolato C, et al.	2020	68	13.6	Other	Research
Predicting early symptomatic osteoarthritis in the human knee using machine learning classification of magnetic resonance images from the Osteoarthritis Initiative [96]	Ashinsky BG, Bouhrara M, Coletta CE, et al.	2017	68	8.5	Other	Outcome prediction
Force and temperature modelling of bone milling using artificial neural networks [97]	Al-Abdullah KIA, Abdi H, Lim CP, Yassin WA	2017	67	8.4	Artificial neural network (ANN)	Research, surgical planning
Computer-aided Cobb measurement based on automatic detection of vertebral slopes using a deep neural network [98]	Zhang J, Li H, Liang Lv, Zhang Y	2017	66	8.3	Artificial neural network (ANN)	Decision support, diagnosis
Evaluating ChatGPT performance on the orthopaedic in-training examination [99]	Kung JE, Marshall C, Gauthier C, Gonzalez TA, Jackson JB	2023	65	32.5	GPT	Research
2020 Frank Stinchfield award: identifying who will fail following irrigation and debridement for prosthetic joint infection [100]	Shohat N, Goswami K, Tan TL, et al.	2020	65	13	Random forest	Outcome prediction
Siam-U-Net: encoder-decoder siamese network for knee cartilage tracking in ultrasound images [101]	Dunnhofer M, Antico M, Sasazawa F, et al.	2020	65	13	Convolutional neural network (CNN), siamese neural network (SNN), U-Net	Image segmentation, surgical planning
Hierarchical fracture classification of proximal femur X-ray images using a multistage deep learning approach [102]	Tanzi L, Vezzetti E, Moreno R, Aprato A, Audisio A, Massè A	2020	63	12.6	Convolutional neural network (CNN), generative adversarial network (GAN)	Diagnosis, decision support, education/training
Experimental evaluation of an adaptive feedforward controller for use in functional neuromuscular stimulation systems [103]	Abbas JJ, Triolo RJ	1997	63	2.3	Artificial neural network (ANN)	Research
Bone fracture detection using deep supervised learning from radiological images: a paradigm shift [104]	Meena T, Roy S	2022	62	20.7	Supervised learning, artificial neural network (ANN), generative adversarial network (GAN)	Diagnosis
Automated system for the detection of thoracolumbar fractures using a CNN architecture [105]	Raghavendra U, Bhat NS, Gudigar A	2018	62	8.9	Convolutional neural network (CNN)	Diagnosis
A review on segmentation of knee articular cartilage: from conventional methods towards deep learning [106]	Ebrahimkhani S, Jaward MH, Cicuttini FM, Dharmaratne A, Wang Y, de Herrera AGS	2020	62	12.4	Convolutional neural network (CNN)	Image segmentation
Exploring the potential of ChatGPT as a supplementary tool for providing orthopaedic information [107]	Kaarre J, Feldt R, Keeling LE, et al.	2023	61	30.5	GPT	Decision support, education/training
Gait phase detection for lower-limb exoskeletons using foot motion data from a single inertial measurement unit in hemiparetic individuals [108]	Sánchez Manchola MDS, Pinto Bernal MJP, Munera M, Cifuentes CA	2019	60	10	Other	Research
A novel method to predict knee osteoarthritis progression on MRI using machine learning methods [109]	Du Y, Almajalid R, Shan J, Zhang M	2018	60	8.6	Supervised vector machine (SVM), random forest, artificial neural network (ANN), and other	Outcome prediction
Filter learning: application to suppression of bony structures from chest radiographs [110]	Loog M, van Ginneken B, Schilham AMR	2006	60	3.2	K-nearest neighbours (KNN)	Image segmentation, diagnosis, decision support, research
A second-order sliding mode control and a neural network to drive. a knee joint actuated orthosis [111]	Mefoued S	2014	59	5.4	Artificial neural network (ANN)	Decision support, outcome analysis
Automatic knee cartilage segmentation from multi-contrast MR images using support vector machine classification with spatial dependencies [112]	Zhang K, Lu W, Marziliano P	2013	59	4.9	Supervised vector machine (SVM), supervised learning	Image segmentation
Artificial intelligence in orthopaedic surgery [113]	Lisacek-Kiosoglous AB, Powling AS, Fontalis A, Gabr A, Mazomenos EB, Haddad FS	2023	58	29	Convolutional neural network (CNN), artificial neural network (ANN)	Research
Is deep learning on par with human observers for the detection of radiographically visible and occult fractures of the scaphoid? [114]	Langerhuizen DWG, Bulstra AEJ, Janssen SJ, et al.	2020	58	11.6	Convolutional neural network (CNN)	Diagnosis
Deep learning-based automated detection of human knee joint's synovial fluid from magnetic resonance images with transfer learning [115]	Iqbal I, Shahzad G, Rafiq N, Mustafa G, Ma J	2020	57	11.4	Convolutional neural network (CNN)	Diagnosis

Publishing Journals and Timestamps

The top-cited articles were distributed across 66 different journals, with the leading contributors presented in Table 2. The Medical Image Analysis journal emerged as the leading contributor, publishing 12 articles. No other journal contributed a double-digit number of publications.. Clinical Orthopaedics and Related Research contributed six articles, while Transactions on Biomedical Engineering and Computerized Medical Imaging and Graphics published five articles each.

**Table 2:** Top 10 journals ranked in descending order by total number of published articles

Journal	Number of published articles
Medical Image Analysis	12
IEEE Transactions on Biomedical Engineering	5
IEEE Transactions on Medical Imaging	4
Clinical Orthopaedics and Related Research	6
Computerized Medical Imaging and Graphics	5
The Spine Journal	3
Journal of Digital Imaging	3
Neurosurgery	2
Journal of Bone and Joint Surgery - American Volume	2
NPJ Digital Medicine	2

**Figure 2:**
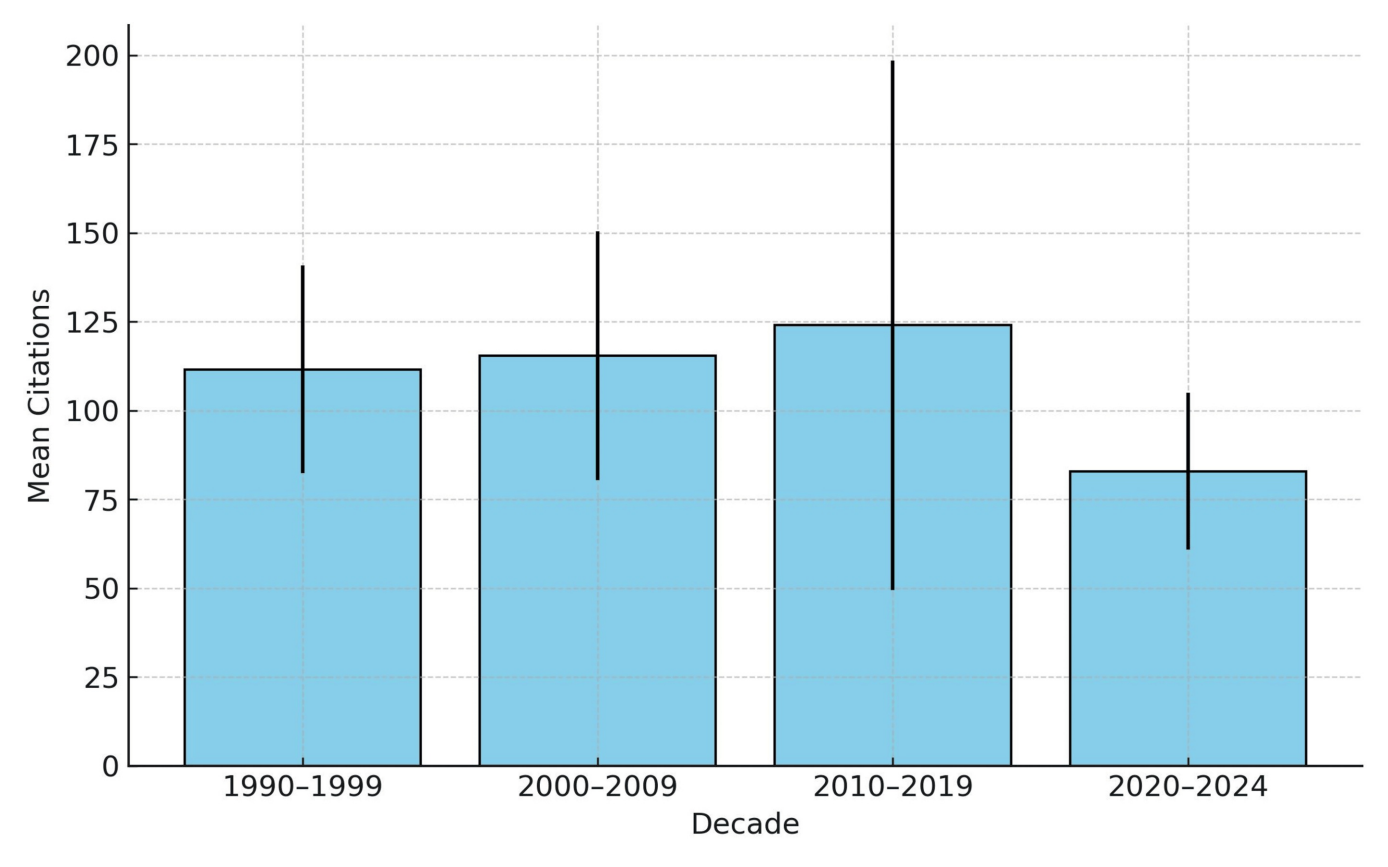
Mean citation count per decade with standard deviation

In terms of cumulative citations per decade, the highest figures were observed in the 2010s (122.4 ± 73.6), followed by the 2000s (116.1 ± 36), as illustrated in Figure 2.

Level of Evidence and Study Designs

The distribution of studies across levels of evidence revealed a predominance of lower-level studies. Level III studies demonstrated the highest average citation count (n = 123.5 ± 87.3), indicating wide variability in citation impact within this group. Further analysis demonstrated that the majority of studies were classified as level of evidence (LOE) V (n = 46; mean citations = 108 ± 41.9), with 43 of them being experimental in terms of study design. A total of 27 studies were retrospective and three were prospective, though only one of them was LOE I, reflecting the paucity of higher-level evidence studies within the field (Tables 3, 4).

**Table 3:** Citation count analysis according to level of evidence

Level of evidence	Number of articles	Mean citations	Standard deviation
I	1	62	N/A
II	8	85.5	20.2
III	24	123.5	87.3
IV	21	107.4	44.5
V	46	108	41.9

**Table 4:** Study design analysis of included studies

Study design	Number of studies
Experimental	43
Retrospective	27
Narrative review	8
Observational	5
Cross-sectional	5
Prospective	3
Systematic review	2
Validation study	2
Expert opinion	2
Case series	1
Computer simulation study	1

Country and Author Trends

The United States of America emerged as by far the most prolific contributing nation, with a total of 45 publications, 21 of which as part of multi-centre studies (n = 43). The Netherlands and the United Kingdom had a notable presence with 12 and 10 publications respectively, with Canada, Australia and Germany contributing nine, eight and five studies respectively. Overall, 32 different countries have contributed to the 100 most cited articles (Table 5).

**Table 5:** Analysis of number of articles by each contributing country

Country	Number of articles
USA	45
Netherlands	12
UK	10
Canada	9
Australia	8
Germany	5
China	5
India	4
France	4
Italy	4
Japan	4
Greece	3
Switzerland	3
Taiwan	3
Sweden	2
Israel	2
Poland	2
Finland	2
Spain	2
Malaysia	2
South Korea	1
Hong Kong	1
Luxembourg	1
Korea	1
Belgium	1
Czech Republic	1
Ireland	1
Portugal	1
Colombia	1
Denmark	1
Singapore	1
Pakistan	1

Among the authors with significant contributions to ML in orthopaedics literature, Karhade emerged as the most prolific overall, having authored a total of five publications, all as first author. Thio and Schwab have also authored a total of five publications, though with one and nil first authorships, respectively. Bono, Ogink, and Schoenfeld have published four papers each, albeit with no first authorships by any.

*Distribution of Articles According to Subspecialty and Anatomical Focus*

Analysis of the articles according to the relevant orthopaedic subspecialty revealed that imaging was the most prevalent focus, accounting for 44 out of 100 studies, representing the emerging nature of ML not only in orthopaedics but also in medicine. A total of 32 articles focused on research and innovation in general, with no specific subspecialty focus, while trauma was represented in 23 articles and arthroplasty in 21 studies. Interestingly, despite the increasing prevalence and subsequent emerging interest around the field of prosthetic joint infections (PJIs), only one study focused on the application of ML in the diagnosis and treatment of such conditions. The knee (n = 42), hip (n = 30), and spine (n = 28) were the most frequently investigated anatomical sites in the included studies.

Machine Learning Techniques, Field of Machine Learning and Task

Among the top 100 most-cited articles in machine learning applied to orthopaedics, CNNs were the most frequently reported technique (n = 37), followed by artificial neural networks (ANNs) (n = 19) and methods categorised as other (n = 15). Traditional statistical and machine learning methods were also common, with random forests (n = 13) and other tree-based ensembles such as extreme gradient boosting (xGB) featuring prominently. Classical statistical models, such as logistic regression, were also represented in seven studies. Support vector machines (SVMs) (n = 11) and statistical shape modelling (SSM) (n = 10) were also well represented, while more advanced generative and deep learning techniques such as GANs and GPT-based approaches appeared in five articles each. Notably, five studies did not provide explicit information on the ML technique employed.

Deep learning was the most frequently discussed ML field, appearing in 53 articles, followed by supervised learning in 42 articles and computer vision in 27. Unsupervised learning, other topics and articles with no specified field were each reported on eight occasions. Generative AI appeared in five articles, natural language processing in three, and robotic AI in two. These findings highlight a predominant focus on deep learning and supervised learning, with emerging areas such as generative AI and robotic AI receiving comparatively limited attention.

The predominant clinical task amongst the top 100 highest cited articles in ML in orthopaedics was diagnosis (43%), with 28% and 27% of articles involving image segmentation and decision support, respectively. Model performance metrics were predominantly standard classification measures, with accuracy reported in 39 articles, area under the curve (AUC) in 22, and sensitivity and specificity in 21 each. Task-specific metrics such as the dice coefficient (n = 16) for image segmentation and root mean square error (n = 15) were less frequently reported, while calibration and localisation metrics were rare (Table 6). 

**Table 6:** Summary of the top 10 most frequently reported ML performance metrics in the top 100 most cited articles relevant to ML in orthopaedics

Model performance metric	Number of articles
Accuracy	39
Area under the curve (AUC)	22
Specificity	21
Sensitivity	21
Dice coefficient	16
Root mean square error (RMSE)	15
Brier score	3
C-statistic	3
Calibration slope	3
Localisation error	2
F1 score	2

Discussion

Advancements and Trends of Machine Learning Applications in Orthopaedics

The ML and AI technologies have made significant progress in medicine and healthcare, creating the recent paradigm shift [116]. Deep learning is being increasingly applied in both medical and surgical fields through implementation in sections such as pre-operative planning optimisation, intra-operative performance amelioration and diagnostic accuracy [117,118]. Analysis of findings from our bibliometric analysis on the application of ML in orthopaedics confirms a strong focus of highly-cited literature on deep and supervised learning, reflecting their effectiveness in handling imaging and structured data. ML performs multiple tasks that aid in image analysis, with the most commonly used tasks being detection, classification and segmentation [119].

Similar to our results, a recent bibliometric analysis of AI in orthopaedics identified algorithmic advancements, applications in disease imaging and innovations in multimodal fusion and 3D imaging techniques as primary scopes of research [120]. It also highlighted a trend towards correlating imaging data with clinical biomarkers to aid decision-making. Our analysis found that highly cited relevant literature has concentrated more heavily on fracture detection and image segmentation tasks. These findings indicate that the orthopaedic community has embraced imaging-related ML applications as the most clinically impactful to date. However, the field will benefit from the implementation of such models in concurrent correlational analysis of multimodal parameters to ensure not only a holistic but also an individualised, evidence-based approach.

*The Expanding Role of Machine Learning in Outcome Prediction*

One area that could particularly benefit from such broader integration is outcome prediction, where ML models have the potential to support personalised prognostication, guide surgical decision-making and optimise postoperative care. ML techniques are unique in their human-reminiscent ability to train, their learning capabilities from the past and their ability to make intelligent decisions [121]. Their future implementation in the field of orthopaedics should aim to train such models so that predictive models are built. For instance, ML could be used to forecast implant survival and the risk of revision following joint replacement, predict postoperative complications such as infection or thromboembolism and estimate functional recovery in terms of gait, pain, or range of motion. Imaging-based models may also support prediction of fracture healing versus progression to non-union, as well as oncological outcomes such as tumour recurrence or metastasis after resection. Finally, as Golberg et al. analysed, the collection of external workload data using ML-driven wearables and sensors can provide significant information regarding functional rehabilitation following ACL reconstruction [122]. As arthroplasty emerges, such models may be implemented to monitor objective rehabilitation outcomes, such as range of motion, gait patterns and adherence to physiotherapy protocols, for post-operative outcome prediction in hip and knee arthroplasty.

Comparison of AI and ChatGPT With Human Expertise

Regarding diagnostic performance, several studies have explored the following contemporary dilemma in medicine: Does AI outperform humans? Shen et al. concluded that current AI development has a diagnostic performance comparable with medical experts, especially in image recognition-related fields, while Jarrahi supported that AI easily outperforms humans in processing, analysing and finding patterns in complex, high-dimensional data [123,124]. In our dataset, 17% of the studies explicitly included a human comparison group in the ML model analysis. Among them, it is found that AI in orthopaedics demonstrates performance approaching, but rarely exceeding, human expert levels (consultant/attending and resident orthopaedic surgeons or radiologists or emergency medicine physicians).

In fracture detection, AI-assisted systems often improved clinician accuracy, yet head-to-head comparisons revealed mixed outcomes: while AI outperformed humans of such expertise in certain fracture types (e.g., hip and proximal humerus), it underperformed in others, such as scaphoid fractures, particularly in specificity and inter-observer agreement. Therefore, the foremost notion that needs to be maintained is that AI remains primarily a supportive tool, rather than a replacement for clinical expertise, with its greatest value in reducing oversight errors or identifying subtle pathology. Furthermore, across the majority of studies, inter-observer reliability among surgeons was consistently higher than algorithm-human agreement, suggesting how critical human oversight is. These findings are consistent with the results of a systematic review by Takita et al., in which AI models performed significantly worse than expert physicians in terms of overall diagnostic performance [125]. 

Similarly, ChatGPT has successfully applied itself in numerous aspects of medicine, including diagnosis, prioritisation, treatment, administration and academia [126]. Direct comparison between this ML model and humans has been previously studied in literature in various of the aforementioned applications [127,128]. Our bibliometric analysis revealed four studies which directly compared ChatGPT (versions 3.5 and 4.0) to orthopaedic doctors. Three of them assessed their performance in USA orthopaedic resident examinations, with the model proving superior to humans on only one occasion. The fourth article assessed ChatGPT’s responses to a quiz related to the anterior cruciate ligament, and 15% of its responses were deemed wrong by senior knee surgeons. These results indicate that, although ChatGPT shows promise in orthopaedic applications, its performance remains variable and currently insufficient to supplant human expertise. This variability likely stems from its training on general language content rather than specialised orthopaedic content, coupled with its inability to emulate the nuanced clinical judgment and experiential knowledge that practising surgeons bring to decision-making. 

Ethical, Legal, and Regulatory Considerations

Integration of AI and ML in healthcare systems raises significant ethical considerations that must be carefully addressed to ensure responsible and equitable deployment [129]. As Ali et al. highlighted in their systematic review regarding the use of AI in the healthcare sector, whilst AI and its subareas provide benefits to individuals, organisations and health sectors, there are some challenges, such as data integration, privacy issues, legal issues and patient safety, which must be carefully addressed [130]. In particular, the implementation of such tools should occur under the guidance of experienced clinicians to ensure appropriate interpretation, patient safety, and adherence to professional standards. Furthermore, the potential bias that may exist within such models can also inadvertently lead to unfair and potentially detrimental outcomes, mainly regarding data, development and interaction bias; hence, adherence to frameworks such as General Data Protection Regulation (GDPR) may be necessary [131].

Interestingly, despite the growing concern of ethical and regulatory bodies regarding the application and transparency of these models, our analysis demonstrated that only 20% of the top-cited articles in ML in orthopaedics successfully incorporated an ethical or regulatory discussion in their manuscripts, indicating that the vast majority of the articles failed to identify the significance of addressing this aspect. This discrepancy likely reflects the field’s primary emphasis on technical performance and clinical validation, with comparatively limited attention given to ethical and regulatory frameworks. Furthermore, researchers may have insufficient familiarity with relevant governance requirements, and given that ML applications in orthopaedics remain in an emergent stage, ethical and regulatory considerations have not yet been consistently incorporated into study design and reporting. This evidently highlights a critical gap in the current literature and underscores the pressing need for greater integration of ethical and regulatory considerations in future research on ML applications within orthopaedics.

Gaps in Evaluation Metrics and Future Research Directions

The current research landscape around ML in orthopaedics is mainly based on traditional metrics such as accuracy, AUC, sensitivity and specificity. On the other hand, task-specific metrics such as dice coefficient for segmentation (a measure of overlap between predicted and actual regions in an image segmentation task, e.g., delineating a bone or joint structure on MRI/CT) are less frequently used. Similarly, calibration metrics that assess the reliability of predicted probabilities and localisation metrics that measure how accurately a model identifies the site of pathology (e.g., fracture lines or implants) remain rare. Subsequently, potential gaps in comprehensive model evaluation and reporting are underscored. Improved model evaluation using more robust metrics could enhance clinical adoption and trustworthiness. In addition, future research within the field should incorporate expansion of ML applications to underexplored tasks, such as surgical planning, outcome prediction and training of resident orthopaedic surgeons. Ideally, this should involve designing studies of a higher level of evidence, such as randomised controlled trials or large-scale prospective validation studies, in order to strengthen the evidence base as well as proactively deal with the current paucity of high-quality prospective studies in the field.

Study Limitations

Bibliometric analyses are inherently susceptible to methodological bias [132]. Bias may arise from institutional or editorial favouritism and self-citation bias [133]. As only English-language publications were included, language bias may have been introduced. Furthermore, Bradford’s “Law of Scattering” posits that literature on a particular subject is predominantly concentrated within a limited number of core journals [134]. Therefore, our search results may have been affected by not identifying articles published in journals of a lower impact factor or journals which are non-indexed. Citation frequency does not necessarily correlate with reporting quality, analytical sophistication or overall study impact [135]. A robust evaluation of individual studies for methodological and data quality is warranted, given that unintentional exclusion of foundational literature may account for some omissions in this analysis. [136,137].

## Conclusions

Future research on ML in orthopaedics should focus on integrating multimodal data and expanding applications to outcome prediction, surgical planning, and resident training. Additionally, such models should be critically appraised with more robust performance metrics and, preferably, within the context of studies of a higher level of evidence. We maintain that advancing a cooperative relationship between AI and human clinicians, rather than pursuing direct comparisons, will best serve to enhance orthopaedic patient outcomes worldwide.

## References

[1] Soori M, Arezoo B, Dastres R (2023). Artificial intelligence, machine learning and deep learning in advanced robotics, a review. Cogn Robot.

[2] Kufel J, Bargieł-Łączek K, Kocot S (2023). What is machine learning, artificial neural networks and deep learning?-Examples of practical applications in medicine. Diagnostics (Basel).

[3] Rehman MU, Naseem S, Butt AU (2025). Predicting coronary heart disease with advanced machine learning classifiers for improved cardiovascular risk assessment. Sci Rep.

[4] Akhtar S, Aftab S, Ali O, Ahmad M, Khan MA, Abbas S, Ghazal TM (2025). A deep learning based model for diabetic retinopathy grading. Sci Rep.

[5] Aydın M, Orhan F (2025). Evaluating the impact of AI in orthopedics: a quantitative analysis of advancements and challenges. Bratisl Med J.

[6] Frid-Adar M, Diamant I, Klang E, Amitai M, Goldberger J, Greenspan H (2018). GAN-based synthetic medical image augmentation for increased CNN performance in liver lesion classification. Neurocomputing.

[7] Farhadi F, Barnes MR, Sugito HR, Sin JM, Henderson ER, Levy JJ (2022). Applications of artificial intelligence in orthopaedic surgery. Front Med Technol.

[8] Han F, Huang X, Wang X (2025). Artificial intelligence in orthopedic surgery: current applications, challenges, and future directions. MedComm (2020).

[9] Nallamothu PT, Bharadiya J (2023). Artificial intelligence in orthopedics: a concise review. Asian J Orthop Res.

[10] Stead WW (2018). Clinical implications and challenges of artificial intelligence and deep learning. JAMA.

[11] (2025). BOA: Integrating artificial intelligence into trauma and orthopaedics: history, current state of AI in T&O and future perspectives. https://www.boa.ac.uk/resource/integrating-artificial-intelligence-into-trauma-and-orthopaedics.html.

[12] Moodley J, Singh V, Kagina BM, Abdullahi L, Hussey GD (2015). A bibliometric analysis of cancer research in South Africa: study protocol. BMJ Open.

[13] Ellegaard O, Wallin JA (2015). The bibliometric analysis of scholarly production: how great is the impact?. Scientometrics.

[14] (2025). Jamovi open statistical software. https://www.jamovi.org.

[15] (2025). OCEBM levels of evidence working group. The Oxford 2011 levels of evidence. Oxford centre for evidence-based medicine. http://www.cebm.net/index.aspx.

[16] Lindsey R, Daluiski A, Chopra S (2018). Deep neural network improves fracture detection by clinicians. Proc Natl Acad Sci U S A.

[17] Kim DH, MacKinnon T (2018). Artificial intelligence in fracture detection: transfer learning from deep convolutional neural networks. Clin Radiol.

[18] Ambellan F, Tack A, Ehlke M, Zachow S (2019). Automated segmentation of knee bone and cartilage combining statistical shape knowledge and convolutional neural networks: data from the Osteoarthritis Initiative. Med Image Anal.

[19] Lessmann N, van Ginneken B, de Jong PA, Išgum I (2019). Iterative fully convolutional neural networks for automatic vertebra segmentation and identification. Med Image Anal.

[20] Yin Y, Zhang X, Williams R, Wu X, Anderson DD, Sonka M (2010). LOGISMOS--layered optimal graph image segmentation of multiple objects and surfaces: cartilage segmentation in the knee joint. IEEE Trans Med Imaging.

[21] Pietka E, Gertych A, Pospiech S, Cao F, Huang HK, Gilsanz V (2001). Computer-assisted bone age assessment: image preprocessing and epiphyseal/metaphyseal ROI extraction. IEEE Trans Med Imaging.

[22] Urakawa T, Tanaka Y, Goto S, Matsuzawa H, Watanabe K, Endo N (2019). Detecting intertrochanteric hip fractures with orthopedist-level accuracy using a deep convolutional neural network. Skeletal Radiol.

[23] Duryea J, Li J, Peterfy CG, Gordon C, Genant HK (2000). Trainable rule-based algorithm for the measurement of joint space width in digital radiographic images of the knee. Med Phys.

[24] Hiasa Y, Otake Y, Takao M (2018). Cross-modality image synthesis from unpaired data using CycleGAN. Lect Notes Comput Sci.

[25] Zhou Z, Zhao G, Kijowski R, Liu F (2018). Deep convolutional neural network for segmentation of knee joint anatomy. Magn Reson Med.

[26] Benameur S, Mignotte M, Parent S, Labelle H, Skalli W, de Guise J (2003). 3D/2D registration and segmentation of scoliotic vertebrae using statistical models. Comput Med Imaging Graph.

[27] Glocker B, Zikic D, Konukoglu E, Haynor DR, Criminisi A (2013). Vertebrae localization in pathological spine CT via dense classification from sparse annotations. Med Image Comput Comput Assist Interv.

[28] Fontana MA, Lyman S, Sarker GK, Padgett DE, MacLean CH (2019). Can machine learning algorithms predict which patients will achieve minimally clinically important differences from total joint arthroplasty?. Clin Orthop Relat Res.

[29] Baka N, Kaptein BL, de Bruijne M, van Walsum T, Giphart JE, Niessen WJ, Lelieveldt BP (2011). 2D-3D shape reconstruction of the distal femur from stereo X-ray imaging using statistical shape models. Med Image Anal.

[30] Abbas JJ, Chizeck HJ (1995). Neural network control of functional neuromuscular stimulation systems: computer simulation studies. IEEE Trans Biomed Eng.

[31] Kostov A, Andrews BJ, Popović DB, Stein RB, Armstrong WW (1995). Machine learning in control of functional electrical stimulation systems for locomotion. IEEE Trans Biomed Eng.

[32] Tang Z, Zhang K, Sun S, Gao Z, Zhang L, Yang Z (2014). An upper-limb power-assist exoskeleton using proportional myoelectric control. Sensors (Basel).

[33] Karhade AV, Thio QC, Ogink PT (2019). Predicting 90-day and 1-year mortality in spinal metastatic disease: development and internal validation. Neurosurgery.

[34] Mavroidis C, Ranky RG, Sivak ML (2011). Patient specific ankle-foot orthoses using rapid prototyping. J Neuroeng Rehabil.

[35] Myers TG, Ramkumar PN, Ricciardi BF, Urish KL, Kipper J, Ketonis C (2020). Artificial intelligence and orthopaedics: an introduction for clinicians. J Bone Joint Surg Am.

[36] Shamir L, Ling SM, Scott WW Jr (2009). Knee x-ray image analysis method for automated detection of osteoarthritis. IEEE Trans Biomed Eng.

[37] Barratt DC, Chan CS, Edwards PJ, Penney GP, Slomczykowski M, Carter TJ, Hawkes DJ (2008). Instantiation and registration of statistical shape models of the femur and pelvis using 3D ultrasound imaging. Med Image Anal.

[38] Otake Y, Armand M, Armiger RS, Kutzer MD, Basafa E, Kazanzides P, Taylor RH (2012). Intraoperative image-based multiview 2D/3D registration for image-guided orthopaedic surgery: incorporation of fiducial-based C-arm tracking and GPU-acceleration. IEEE Trans Med Imaging.

[39] Ghaednia H, Fourman MS, Lans A (2021). Augmented and virtual reality in spine surgery, current applications and future potentials. Spine J.

[40] Kauffmann C, Gravel P, Godbout B (2003). Computer-aided method for quantification of cartilage thickness and volume changes using MRI: validation study using a synthetic model. IEEE Trans Biomed Eng.

[41] Michopoulou SK, Costaridou L, Panagiotopoulos E, Speller R, Panayiotakis G, Todd-Pokropek A (2009). Atlas-based segmentation of degenerated lumbar intervertebral discs from MR images of the spine. IEEE Trans Biomed Eng.

[42] Lee S, Park M, Lee K, Lee J (2019). Scalable muscle-actuated human simulation and control. ACM Trans Graph.

[43] Wu H, Bailey C, Rasoulinejad P, Li S (2018). Automated comprehensive adolescent idiopathic scoliosis assessment using MVC-Net. Med Image Anal.

[44] Li X, Dou Q, Chen H (2018). 3D multi-scale FCN with random modality voxel dropout learning for intervertebral disc localization and segmentation from multi-modality MR Images. Med Image Anal.

[45] Benameur S, Mignotte M, Parent S, Labelle H, Skalli W, de Guise J (2003). 3D/2D registration and segmentation of scoliotic vertebrae using statistical models. Comput Med Imaging Graph.

[46] Kaus MR, Pekar V, Lorenz C, Truyen R, Lobregt S, Weese J (2003). Automated 3-D PDM construction from segmented images using deformable models. IEEE Trans Med Imaging.

[47] Condino S, Turini G, Parchi PD (2018). How to build a patient-specific hybrid simulator for orthopaedic open surgery: benefits and limits of mixed-reality using the Microsoft HoloLens. J Healthc Eng.

[48] Langerhuizen DW, Janssen SJ, Mallee WH (2019). What are the applications and limitations of artificial intelligence for fracture detection and classification in orthopaedic trauma imaging? A systematic review. Clin Orthop Relat Res.

[49] Mika AP, Martin JR, Engstrom SM, Polkowski GG, Wilson JM (2023). Assessing ChatGPT responses to common patient questions regarding total hip arthroplasty. J Bone Joint Surg Am.

[50] Karhade AV, Thio QC, Ogink PT (2019). Development of machine learning algorithms for prediction of 30-day mortality after surgery for spinal metastasis. Neurosurgery.

[51] Stindel E, Briard JL, Merloz P, Plaweski S, Dubrana F, Lefevre C, Troccaz J (2002). Bone morphing: 3D morphological data for total knee arthroplasty. Comput Aided Surg.

[52] Chang GC, Lub JJ, Liao GD (1997). A neuro-control system for the knee joint position control with quadriceps stimulation. IEEE Trans Rehabil Eng.

[53] Kalmet PH, Sanduleanu S, Primakov S (2020). Deep learning in fracture detection: a narrative review. Acta Orthop.

[54] Ley C, Martin RK, Pareek A, Groll A, Seil R, Tischer T (2022). Machine learning and conventional statistics: making sense of the differences. Knee Surg Sports Traumatol Arthrosc.

[55] Sadik M, Hamadeh I, Nordblom P, Suurkula M, Höglund P, Ohlsson M, Edenbrandt L (2008). Computer-assisted interpretation of planar whole-body bone scans. J Nucl Med.

[56] Rosen J, Fuchs MB, Arcan M (1999). Performances of hill-type and neural network muscle models-toward a myosignal-based exoskeleton. Comput Biomed Res.

[57] Norman B, Pedoia V, Noworolski A, Link TM, Majumdar S (2019). Applying densely connected convolutional neural networks for staging osteoarthritis severity from plain radiographs. J Digit Imaging.

[58] Wang L, Chen X, Deng X (2024). Prompt engineering in consistency and reliability with the evidence-based guideline for LLMs. NPJ Digit Med.

[59] Zhang A, Gertych A, Liu BJ (2007). Automatic bone age assessment for young children from newborn to 7-year-old using carpal bones. Comput Med Imaging Graph.

[60] Chang PD, Wong TT, Rasiej MJ (2019). Deep learning for detection of complete anterior cruciate ligament tear. J Digit Imaging.

[61] Fleute M, Lavallée S, Julliard R (1999). Incorporating a statistically based shape model into a system for computer-assisted anterior cruciate ligament surgery. Med Image Anal.

[62] Cao R, Nelson SD, Davis S (2023). Label-free intraoperative histology of bone tissue via deep-learning-assisted ultraviolet photoacoustic microscopy. Nat Biomed Eng.

[63] Hsieh CI, Zheng K, Lin C (2021). Automated bone mineral density prediction and fracture risk assessment using plain radiographs via deep learning. Nat Commun.

[64] Smyth PP, Taylor CJ, Adams JE (1999). Vertebral shape: automatic measurement with active shape models. Radiology.

[65] Karhade AV, Ogink PT, Thio QC (2019). Machine learning for prediction of sustained opioid prescription after anterior cervical discectomy and fusion. Spine J.

[66] Neubert A, Fripp J, Engstrom C, Schwarz R, Lauer L, Salvado O, Crozier S (2012). Automated detection, 3D segmentation and analysis of high resolution spine MR images using statistical shape models. Phys Med Biol.

[67] Subburaj K, Ravi B, Agarwal M (2009). Automated identification of anatomical landmarks on 3D bone models reconstructed from CT scan images. Comput Med Imaging Graph.

[68] Jun K, Lee DW, Lee K, Lee S, Kim MS (2020). Feature extraction using an RNN autoencoder for skeleton-based abnormal gait recognition. IEEE Access.

[69] Jones RM, Sharma A, Hotchkiss R (2020). Assessment of a deep-learning system for fracture detection in musculoskeletal radiographs. NPJ Digit Med.

[70] Al Arif SM, Knapp K, Slabaugh G (2018). Fully automatic cervical vertebrae segmentation framework for X-ray images. Comput Methods Programs Biomed.

[71] Massey PA, Montgomery C, Zhang AS (2023). Comparison of ChatGPT-3.5, ChatGPT-4, and orthopaedic resident performance on orthopaedic assessment examinations. J Am Acad Orthop Surg.

[72] Lum ZC (2023). Can artificial intelligence pass the American Board of Orthopaedic Surgery examination? Orthopaedic residents versus ChatGPT. Clin Orthop Relat Res.

[73] Schmid J, Kim J, Magnenat-Thalmann N (2011). Robust statistical shape models for MRI bone segmentation in presence of small field of view. Med Image Anal.

[74] Rangayyan RM, Wu YF (2008). Screening of knee-joint vibroarthrographic signals using statistical parameters and radial basis functions. Med Biol Eng Comput.

[75] Mamo HB, Adamiak M, Kunwar A (2023). 3D printed biomedical devices and their applications: a review on state-of-the-art technologies, existing challenges, and future perspectives. J Mech Behav Biomed Mater.

[76] Kokkotis C, Moustakidis S, Papageorgiou E, Giakas G, Tsaopoulos DE (2020). Machine learning in knee osteoarthritis: a review. Osteoarthr Cartil Open.

[77] Zoroofi RA, Sato Y, Sasama T (2003). Automated segmentation of acetabulum and femoral head from 3-D CT images. IEEE Trans Inf Technol Biomed.

[78] Karhade AV, Ogink PT, Thio QC (2019). Development of machine learning algorithms for prediction of prolonged opioid prescription after surgery for lumbar disc herniation. Spine J.

[79] Liu P, Han H, Du Y (2021). Deep learning to segment pelvic bones: large-scale CT datasets and baseline models. Int J Comput Assist Radiol Surg.

[80] Tiulpin A, Saarakkala S (2020). Automatic grading of individual knee osteoarthritis features in plain radiographs using deep convolutional neural networks. Diagnostics (Basel).

[81] Audenaert EA, Van Houcke J, Almeida DF, Paelinck L, Peiffer M, Steenackers G, Vandermeulen D (2019). Cascaded statistical shape model based segmentation of the full lower limb in CT. Comput Methods Biomech Biomed Engin.

[82] Huber M, Kurz C, Leidl R (2019). Predicting patient-reported outcomes following hip and knee replacement surgery using supervised machine learning. BMC Med Inform Decis Mak.

[83] Cheng CT, Wang Y, Chen HW (2021). A scalable physician-level deep learning algorithm detects universal trauma on pelvic radiographs. Nat Commun.

[84] Kirchner GJ, Kim RY, Weddle JB, Bible JE (2023). Can artificial intelligence improve the readability of patient education materials?. Clin Orthop Relat Res.

[85] Chmelik J, Jakubicek R, Walek P (2018). Deep convolutional neural network-based segmentation and classification of difficult to define metastatic spinal lesions in 3D CT data. Med Image Anal.

[86] Lee JG, Gumus S, Moon CH, Kwoh CK, Bae KT (2014). Fully automated segmentation of cartilage from the MR images of knee using a multi-atlas and local structural analysis method. Med Phys.

[87] Thio QC, Karhade AV, Ogink PT, Raskin KA, De Amorim Bernstein K, Lozano Calderon SA, Schwab JH (2018). Can machine-learning techniques be used for 5-year survival prediction of patients with chondrosarcoma?. Clin Orthop Relat Res.

[88] Cao Y, Huang J (2020). Neural-network-based nonlinear model predictive tracking control of a pneumatic muscle actuator-driven exoskeleton. IEEE/CAA J Autom Sin.

[89] Mutasa S, Varada S, Goel A, Wong TT, Rasiej MJ (2020). Advanced deep learning techniques applied to automated femoral neck fracture detection and classification. J Digit Imaging.

[90] Hetherington J, Lessoway V, Gunka V, Abolmaesumi P, Rohling R (2017). SLIDE: automatic spine level identification system using a deep convolutional neural network. Int J Comput Assist Radiol Surg.

[91] Fitzpatrick CK, Baldwin MA, Laz PJ, FitzPatrick DP, Lerner AL, Rullkoetter PJ (2011). Development of a statistical shape model of the patellofemoral joint for investigating relationships between shape and function. J Biomech.

[92] Kozic N, Weber S, Büchler P, Lutz C, Reimers N, González Ballester MA, Reyes M (2010). Optimisation of orthopaedic implant design using statistical shape space analysis based on level sets. Med Image Anal.

[93] Subburaj K, Ravi B, Agarwal M (2009). Automated 3D geometric reasoning in computer-assisted joint reconstructive surgery. Proc 2009 IEEE Int Conf Autom Sci Eng.

[94] Pietka E, Pospiech-Kurkowska S, Gertych A, Cao F (2003). Integration of computer assisted bone age assessment with clinical PACS. Comput Med Imaging Graph.

[95] Saxby DJ, Killen BA, Pizzolato C (2020). Machine learning methods to support personalized neuromusculoskeletal modelling. Biomech Model Mechanobiol.

[96] Ashinsky BG, Bouhrara M, Coletta CE (2017). Predicting early symptomatic osteoarthritis in the human knee using machine learning classification of magnetic resonance images from the osteoarthritis initiative. J Orthop Res.

[97] Al-Abdullah KIA, Abdi H, Lim CP, Yassin WA (2018). Force and temperature modelling of bone milling using artificial neural networks. Measurement.

[98] Zhang J, Li H, Lv L, Zhang Y (2017). Computer-aided Cobb measurement based on automatic detection of vertebral slopes using deep neural network. Int J Biomed Imaging.

[99] Kung JE, Marshall C, Gauthier C, Gonzalez TA, Jackson JB 3rd (2023). Evaluating ChatGPT performance on the orthopaedic in-training examination. JB JS Open Access.

[100] Shohat N, Goswami K, Tan TL (2020). 2020 Frank Stinchfield Award: identifying who will fail following irrigation and debridement for prosthetic joint infection. Bone Joint J.

[101] Dunnhofer M, Antico M, Sasazawa F (2020). Siam-U-Net: encoder-decoder siamese network for knee cartilage tracking in ultrasound images. Med Image Anal.

[102] Tanzi L, Vezzetti E, Moreno R, Aprato A, Audisio A, Massè A (2020). Hierarchical fracture classification of proximal femur X-ray images using a multistage deep learning approach. Eur J Radiol.

[103] Abbas JJ, Triolo RJ (1997). Experimental evaluation of an adaptive feedforward controller for use in functional neuromuscular stimulation systems. IEEE Trans Rehabil Eng.

[104] Meena T, Roy S (2022). Bone fracture detection using deep supervised learning from radiological images: a paradigm shift. Diagnostics (Basel).

[105] Raghavendra U, Bhat NS, Gudigar A (2018). Acharya UR: automated system for the detection of thoracolumbar fractures using a CNN architecture. Future Gener Comput Syst.

[106] Ebrahimkhani S, Jaward MH, Cicuttini FM, Dharmaratne A, Wang Y, de Herrera AG (2020). A review on segmentation of knee articular cartilage: from conventional methods towards deep learning. Artif Intell Med.

[107] Kaarre J, Feldt R, Keeling LE (2023). Exploring the potential of ChatGPT as a supplementary tool for providing orthopaedic information. Knee Surg Sports Traumatol Arthrosc.

[108] Sánchez Manchola MD, Pinto Bernal MJ, Munera M, Cifuentes CA (2019). Gait phase detection for lower-limb exoskeletons using foot motion data from a single inertial measurement unit in hemiparetic individuals. Sensors (Basel).

[109] Du Y, Almajalid R, Shan J, Zhang M (2018). A novel method to predict knee osteoarthritis progression on MRI using machine learning methods. IEEE Trans Nanobioscience.

[110] Loog M, van Ginneken B, Schilham AM (2006). Filter learning: application to suppression of bony structures from chest radiographs. Med Image Anal.

[111] Mefoued S (2015). A second order sliding mode control and a neural network to drive a knee joint actuated orthosis. Neurocomputing.

[112] Zhang K, Lu W, Marziliano P (2013). Automatic knee cartilage segmentation from multi-contrast MR images using support vector machine classification with spatial dependencies. Magn Reson Imaging.

[113] Lisacek-Kiosoglous AB, Powling AS, Fontalis A, Gabr A, Mazomenos E, Haddad FS (2023). Artificial intelligence in orthopaedic surgery. Bone Joint Res.

[114] Langerhuizen DW, Bulstra AE, Janssen SJ, Ring D, Kerkhoffs GM, Jaarsma RL, Doornberg JN (2020). Is deep learning on par with human observers for detection of radiographically visible and occult fractures of the scaphoid?. Clin Orthop Relat Res.

[115] Iqbal I, Shahzad G, Rafiq N, Mustafa G, Ma J (2020). Deep learning-based automated detection of human knee joint's synovial fluid from magnetic resonance images with transfer learning. IET Image Process.

[116] Chakraborty C, Bhattacharya M, Pal S, Lee S (2023). From machine learning to deep learning: An advances of the recent data-driven paradigm shift in medicine and healthcare. Curr Res Biotechnol.

[117] Morris MX, Rajesh A, Asaad M, Hassan A, Saadoun R, Butler CE (2023). Deep learning applications in surgery: current uses and future directions. Am Surg.

[118] Aggarwal R, Sounderajah V, Martin G (2021). Diagnostic accuracy of deep learning in medical imaging: a systematic review and meta-analysis. NPJ Digit Med.

[119] Wang VM, Cheung CA, Kozar AJ, Huang B (2020). Machine learning applications in orthopedic imaging. J Am Acad Orthop Surg.

[120] Huang X, Han F, Chen YF (2025). Bibliometric analysis of the application of artificial intelligence in orthopedic imaging. Quant Imaging Med Surg.

[121] Sarker IH (2021). Machine learning: algorithms, real-world applications and research directions. SN Comput Sci.

[122] Golberg E, Pinkoski A, Beaupre L, Rouhani H (2023). Monitoring external workload with wearable technology after anterior cruciate ligament reconstruction: a scoping review. Orthop J Sports Med.

[123] Shen J, Zhang CJ, Jiang B (2019). Artificial intelligence versus clinicians in disease diagnosis: systematic review. JMIR Med Inform.

[124] Jarrahi MH (2018). Artificial intelligence and the future of work: human-AI symbiosis in organizational decision making. Bus Horiz.

[125] Takita H, Kabata D, Walston SL (2025). A systematic review and meta-analysis of diagnostic performance comparison between generative AI and physicians. NPJ Digit Med.

[126] Tan S, Xin X, Wu D (2024). ChatGPT in medicine: prospects and challenges: a review article. Int J Surg.

[127] Zaboli A, Brigo F, Sibilio S, Mian M, Turcato G (2024). Human intelligence versus Chat-GPT: who performs better in correctly classifying patients in triage?. Am J Emerg Med.

[128] Arvidsson R, Gunnarsson R, Entezarjou A, Sundemo D, Wikberg C (2024). ChatGPT (GPT-4) versus doctors on complex cases of the Swedish family medicine specialist examination: an observational comparative study. BMJ Open.

[129] Harishbhai Tilala M, Kumar Chenchala P, Choppadandi A, Kaur J, Naguri S, Saoji R, Devaguptapu B (2024). Ethical considerations in the use of artificial intelligence and machine learning in health care: a comprehensive review. Cureus.

[130] Ali O, Abdelbaki W, Shrestha A, Elbasi E, Alryalat MAA, Dwivedi YK (2023). A systematic literature review of artificial intelligence in the healthcare sector: benefits, challenges, methodologies, and functionalities. J Innov Knowl.

[131] Hanna MG, Pantanowitz L, Jackson B (2025). Ethical and bias considerations in artificial intelligence/machine learning. Mod Pathol.

[132] Dumont JE (1989). The bias of citations. Trends Biochem Sci.

[133] Campbell FM (1990). National bias: a comparison of citation practices by health professionals. Bull Med Libr Assoc.

[134] Alabi G (1979). Bradford’s law and its application. Int Libr Rev.

[135] Nieminen P, Carpenter J, Rucker G, Schumacher M (2006). The relationship between quality of research and citation frequency. BMC Med Res Methodol.

[136] Garfield E (1987). 100 citation classics from the Journal of the American Medical Association. JAMA.

[137] Baltussen A, Kindler CH (2004). Citation classics in anesthetic journals. Anesth Analg.

